# Effects of an Enhanced Training on Primary Care Providers Knowledge, Attitudes, Service and Skills of Dementia Detection: A Cluster Randomized Trial

**DOI:** 10.3389/fneur.2021.651826

**Published:** 2021-07-23

**Authors:** Xiaozhen Lv, Mei Zhao, Tao Li, Changzheng Yuan, Haifeng Zhang, Chengcheng Pu, Zhiying Li, Na Zhang, Xin Yu, Huali Wang

**Affiliations:** ^1^Beijing Dementia Key Lab, Dementia Care & Research Center, Peking University Institute of Mental Health (Sixth Hospital), Beijing, China; ^2^NHC Key Laboratory of Mental Health, National Clinical Research Center for Mental Disorders, Beijing, China; ^3^Academic Unit for Psychiatry of Old Age, Department of Psychiatry, The University of Melbourne, Parkville, VIC, Australia; ^4^Department of Big Data in Health Science, School of Public Health, School of Medicine, Zhejiang University, Hangzhou, China; ^5^Psychological Department, Beijing Anzhen Hospital, Capital Medical University, Beijing, China

**Keywords:** dementia detection, primary care providers, training, knowledge, attitudes, service, cluster randomized trial

## Abstract

**Background:** Effective training programs for primary care providers (PCPs) to support dementia detection are needed, especially in developing countries. This study aimed to investigate the effect of an enhanced training on the competency and service of PCPs for dementia detection.

**Methods:** We conducted a cluster randomized trial in Beijing, China. Community healthcare centers (CHCs) located in Fengtai or Fangshan District were eligible. The enrolled CHCs in each district were randomly assigned to the standard or the enhanced training group at a 1:1 ratio. PCPs serving older adults in enrolled CHCs were eligible to participate. The standard training group received three-hour didactic lectures, three monthly supervisions, 3 months of online support and dementia screening packages. The enhanced training group additionally received three monthly face-to-face supervisions and 3 months of online support. The participants became aware of their group membership at the end of the standard training. The knowledge, attitudes, service, and skills regarding dementia detection were assessed using questionnaires and submitted dementia detection records, respectively.

**Results:** A total of 23 and 21 CHCs were randomly assigned to the standard and the enhanced training group, respectively, and 58 participants from 20 CHCs assigned to the standard training group and 48 from 16 CHCs assigned to the enhanced training group were included in the final analysis (mean age 37.5 years, and 67.0% women). A significant increase in the knowledge score was found in both groups, but the increase was similar in the two groups (*P* = 0.262). The attitude score remained stable in both groups, and no between-group difference was found. Compared with the baseline, both groups reported an increase in dementia detection service, especially the enhanced training group (24.1% to 31.0% in the standard training group and 14.6% to 45.8% in the enhanced training group). The completion rate and accuracy of submitted dementia detection records in the enhanced training group were both significantly higher than those in the standard training group (both *P* < 0.001).

**Conclusion:** The enhanced training had similar effect on the knowledge of PCPs comparing with the standard training, but was better on continuous service and skills of PCPs related to dementia detection.

**Trial registration:** www.ClinicalTrials.gov, identifier: NCT02782000. Registration date: May 2016. The trial was completed in July 2017.

## Introduction

Timely dementia detection is critical if dementia patients are to receive effective interventions and plan accordingly (1, 2). Primary care providers (PCPs) are usually the first clinicians to whom older adults report dementia symptoms; therefore, they are on the front line in timely dementia detection (1, 3, 4). Many PCPs have limited knowledge (e.g., do not recognize early signs of dementia), negative attitudes (e.g., doubtful about the benefits of early diagnosis and treatment) and a lack of skills (e.g., not familiar with dementia screening tools) related to dementia detection in most countries and regions (5–7), which prevents them from detecting and managing dementia in a timely manner (4, 8, 9). Therefore, improving the competency of PCPs regarding dementia detection is imperative worldwide, especially in China, due to its large number of older adults and dementia patients (10, 11), high rate (93.1%) of undetected dementia (12) and limited education and training on dementia detection for PCPs (13).

Training of PCPs plays an important role in timely dementia detection. Previous studies have shown some positive effects of educational intervention on PCPs' knowledge, attitudes, competence, confidence, practice and health care outcomes regarding dementia detection but there is no consistency across studies (14–18). Caution is needed to apply these results to other settings due to the heterogeneity of the intervention components, training methods, expertise of trainees and trainers and methodological robustness across these studies (14, 15). Moreover, the majority of evidence has originated from developed regions, and the required training content and feasible delivery methods in developing countries may differ from those in developed countries (19). To improve the competency of PCPs in dementia detection in developing countries, an effective and feasible dementia detection training program for PCPs is urgently needed.

Our previous pilot study revealed that PCPs' knowledge regarding dementia detection and self-reported dementia screening practice improved after training (20). To provide more reliable evidence on this topic, we conducted the present trial involving 44 community health service centers (CHCs) in Beijing, China. A cluster randomization was chosen for practical reasons and to prevent contamination by communication among PCPs. It aimed to evaluate the effect of an enhanced training on the knowledge, attitudes, provided dementia screening service and skills related to dementia detection of PCPs and to explore the satisfaction and reactions of trainees to the training.

## Materials and Methods

### Study Design

This study was a cluster randomized trial conducted in CHCs in Fangshan and Fengtai Districts, Beijing, China between August 2016 and July 2017. Little dementia detection training was provided in these areas. CHCs are the primary care units of the national health system, and are the foundation of providing primary health care services to residents who are living in their jurisdictions. Collaborating with the Fangshan and Fengtai Health Commissions, the CHCs in these two districts, as long as the leader of the CHC agreed to participate the program, were all eligible. We enrolled 24 of 24 CHCs in Fangshan District and 20 of 23 CHCs in Fengtai District.

The enrolled CHCs in each district were randomly assigned at a 1:1 ratio to the standard or the enhanced training group using a series of computer-generated random numbers by a researcher. The present trial consisted of two phases lasting for 6 months. In the first phase, both the standard and the enhanced training group received three-hour didactic lectures, three monthly face-to-face supervisions, and 3 months of online support and dementia screening toolkits. In the second phase, the enhanced training group received an additional three monthly supervisions and 3 months of online support, whereas the standard training group received no more training or support, except dementia screening toolkits. The participants became aware of their group membership at the end of the first phase.

This trial was conducted with the approval of the Ethics Committee of Peking University Sixth Hospital (2016-Lunshen-7). All participants provided written informed consent after the randomization of the enrolled CHCs.

### Participants

After agreeing to attend the program, the leader of each enrolled center sent the information of the training program to the PCPs. The PCPs working at these selected sites were invited to participate in the study and attended voluntarily. The participant inclusion criteria were as follows: (1) the majority of their patients were older adults; (2) they had access to WeChat (the most popular social media platform in China, which supports online live chats and classrooms for reviewing slides); and (3) they continued working at their current institution for at least 6 months after enrollment. The exclusion criteria were as follows: (1) refusal to complete the questionnaire for assessing the training effect and (2) involvement in another dementia-related study. The final number of participants was determined by the leader of each CHC.

### Intervention

Based on previous studies of dementia detection training (3, 19) and our pilot study (20), a multidisciplinary expert team of geriatric psychiatrists (XY and HW), experts in the field of dementia prevention (HW, TL and XL) and educators (HW and TL) developed the training program that included didactic lectures, supervisions, online support and dementia screening packages to train and support PCPs involved in dementia detection. All four trainers (TL, CP, ZL and NZ) were registered psychiatrists with experience in dementia detection, diagnosis, treatment and education. Before the intervention, the supervisor of the program (HW) reviewed all training slides, and a special workshop for trainers was held to unify the training format.

In the first phase, two groups both received standard training consisting of (1) didactic lectures and exercises: we informed the participants about the prevalence, symptoms, prevention, detection, treatment and referral of dementia through a 50-min lecture, and then we introduced two dementia screening tools [Eight-item Interview to Differentiate Aging and Dementia (AD8) and Clock Drawing Test (CDT)] through a 35-min lecture and guided participants in exercises based on the screening tools; (2) three monthly face-to-face supervisions: the first supervision was provided 1 month after the lectures, and another two were subsequently provided. Each supervision lasted approximately 90 min and followed a uniform format, which was organized into 3 components: review of the key points of the last lecture, a 45-min lecture on dementia symptoms/diagnosis procedure/standard treatment, and case analysis and discussion; (3) 3 months of online support: using the WeChat application, the training slide was uploaded to the online classroom after each lecture for participants to review at any time; in addition, we provided online counseling support about training content, dementia screening and referral in real time through an online discussion group in WeChat or by telephone; and (4) a screening package: a dementia screening package, including training materials, dementia screening and referral records, a referral information card and educational material about dementia for the public, was offered to each trainee at the beginning of the intervention, and these materials were replenished as needed during the study.

In the second phase, the enhanced training group continued receiving three monthly supervisions (in a similar format to that of the first three supervisions, with the lecture introducing dementia differential diagnosis/treatment safety/prevention progress) and 3 months of online support during this period. The standard training group did not receive any intervention during this period, except for the screening toolkit.

The lectures and supervisions were provided at the Center for Continuing Medical Education of each district. During supervision periods, the participants in the included centers, which were assigned to the same group and located in the same district (Fangshan or Fengtai District), received supervision simultaneously. All interventions and procedures for participants from Fangshan District were repeated for those from Fengtai District. To improve their adherence, we provided all participants an opportunity to obtain continuing medical education credit points for attending the training, which was mandatory for all medical workers in China. No other reimbursement was offered for participating in the trial or providing dementia screening services.

### Outcome Assessment

Outcome measures were classified according to Kirkpatrick's model for the evaluation of training interventions (21): (1) the participants' knowledge about, attitudes toward and skills in dementia screening; (2) dementia screening and referral service provided by the participants; and (3) participants' satisfaction with and reaction to the program.

The knowledge score, as the primary outcome, consisted of theoretical and practical knowledge, and was assessed using 13 true-false questions, 13 multiple-choice questions (including the etiology, symptoms, screening, diagnosis, treatment, prevention, prognosis, and referral of dementia), and three case analyses (about the evaluation and referral of suspected dementia patients). This score ranged from 0 to 45, with higher scores denoting greater knowledge.

Attitude was assessed using 10 true-false questions about dementia screening and referral, with scores ranging from 0 to 10, and higher scores denoting a more positive attitude. The dementia screening and referral of patients with suspected dementia provided by the participants was assessed by asking whether they provided these services in the past month, and the number of dementia screening and referral records submitted by PCPs at the end of the trial.

Skills in dementia screening and referral were assessed according to the submitted records, with the completion rate and accuracy of the AD8 and CDT, and the referral suggestions. The completion rates of the AD8, CDT and the referral suggestions were calculated with the number of completed AD8s (all items and total score of AD8 completed), CDTs (total score graded) and the referral suggestions (suggestion given) divided by the number of submitted records, respectively. The accuracy of the AD8s was assessed by the number of AD8s with the correct total score (comparing the total score given by the participants with the total score calculated based on all items) divided by the number of completed AD8s. Similarly, the accuracy of the CDTs was calculated as the number of CDTs with the correct total score (comparing the total score graded by the participants with the total score graded by researchers based on the figure drawn by the screening object) divided by the number of completed CDTs. The accuracy of referral suggestions was assessed according to the number of correct referral suggestions (comparing the referral suggestions given by the participants with the recommended suggestions based on the AD8 and CDT total scores) divided by the number of completed management suggestions.

The satisfaction and reaction of the participants were evaluated using a separate survey, which addressed whether the training program was useful for their clinical practice, their advice for improving the training program, and the advantages and disadvantages of dementia detection in their practice.

All the assessments were examined in exactly the same way for the two groups. Except for the participants' skills and feedback evaluated at the end of the trial, other assessments were conducted at baseline and at the end of the trial. Demographic characteristics of the participants, including sex, age, occupation, education and years of working, were collected at baseline. The questionnaire used for assessing the knowledge, attitudes and self-reported service was shown in supplementary The Questionnaire.

### Sample Size

According to our previous pilot study (20), we estimated that at a 1:1 ratio, with a sample of 32 clusters (CHCs) with an average of 4 participants in each cluster, the study would have 95% power to detect a difference of 3 points (standard deviation of 4) in knowledge score (the primary outcome) between the two groups, with an intracluster correlation of 0.05 and a two-sided significance level of 0.05. Considering up to a 15% loss to follow-up, the number of clusters was increased to 38, and 152 participants were needed.

### Statistical Analysis

The data are presented as the means and standard deviations, medians and interquartile ranges, and counts and percentages for normally distributed variables, non normally distributed variables, and categorical variables, respectively. Between-group differences in baseline characteristics were analyzed with Student's *t* test or the Wilcoxon rank-sum test for continuous variables and chi-square test or Fisher's exact test for categorical variables.

Modified ITT analyses were conducted. Analyses of the difference between the two groups in the change in knowledge and attitude scores and dementia screening and referral between baseline and the end of the intervention were performed on data from the participants who underwent the assessments both at baseline and at the end of the intervention, no matter whether they completed the entire intervention. Analysis of dementia screening and referral skills was performed based on the submitted screening records at the end of the trial. Analysis of the satisfaction and reaction of the participants was conducted on data from the participants who completed the survey at the end of the intervention.

We used paired *t* tests to examine the changes in the knowledge score and attitude score before and after the intervention in each group, respectively. We used paired chi-square tests to examine the proportion of dementia screening and referral provided by the participants before and after the intervention in each group, respectively. We used a generalized linear mixed model to examine the differences in the change in knowledge score, attitude score and the proportion of provided dementia screening and referral of the two groups between baseline and the end of the intervention. Cluster (CHC) was treated as a random effect, and intervention, measure time, and intervention-measure time interaction were treated as fixed effects, with age as a covariate. To evaluate the need to adjust for site, education, occupation and working experience, models included a term for the interaction with the study group (the standard or the enhanced training group). A *P* < 0.05 was considered to indicate a significant interaction. Since all the *P* > 0.15 for interaction, the final model did not adjust for site, education, occupation or working experience. We used chi-square tests to examine the differences in dementia screening and referral skills between the two groups. The participants' reaction was thematically analyzed.

A two-sided *P* < 0.05 was considered statistically significant. All analyses were performed with SAS 9.4.

## Results

### Participant Flow and Baseline Characteristics

**Figure 1:**
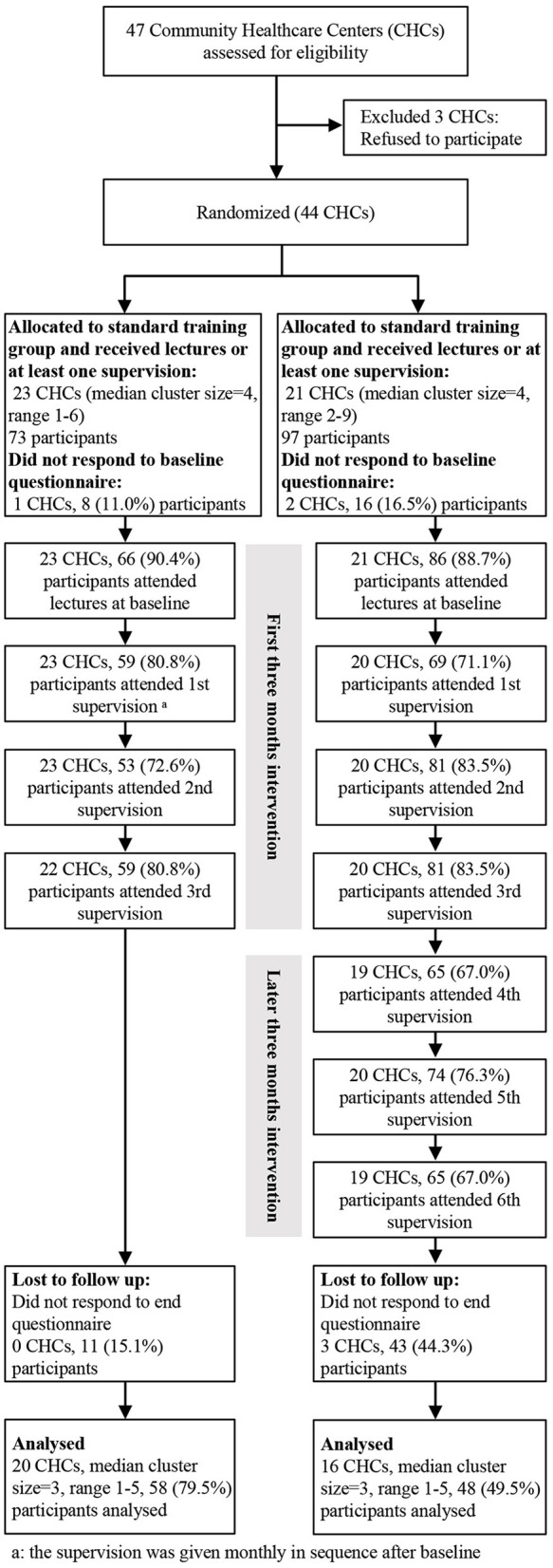
Flow diagram of the sample frame and data collection.

The recruitment started in July 2016 and follow-up ended in July 2017 at the end of the trial. A total of 44 out of 47 CHCs in Fangshan and Fengtai Districts agreed to participate in the trial, and 23 and 21 CHCs were assigned to the standard and enhanced training groups, respectively. Initially, 73 PCPs from the 23 CHCs in the standard training group and 97 from the 21 CHCs in the enhanced training group entered the trial. During the intervention, the attrition rates in the standard and enhanced training group were 18.8% and 23.3%, respectively, and no between-group difference was found (*P* = 0.126). At the end of the trial, participants in the enhanced training group were more likely lost to follow-up than those in the standard training group (44.3 vs. 15.1%). Finally, a total of 58 (79.5%) participants from 20 CHCs in the standard training group and 48 (49.5%) from 16 CHCs in the enhanced training group completed both the baseline and the last assessment and were included in the analysis (Figure 1).

The median (P_25_-P_75_) cluster size was 3 (2–4). The average age of the participants was 37.5 years, and 67.0% were female. The majority of the participants were physicians (73.6%) and had a bachelor's degree or above (66.6%). The median (P_25_-P_75_) years of working was 10 (6–16). Except for age, the characteristics of the participants between the two groups did not differ significantly (Table 1). The characteristics of the participants lost to follow-up were similar to those included in the analysis; the except was education, namely, those with lower education level were more likely to be lost to follow-up than those with higher education level (*P* = 0.006). The CONSORT 2010 checklist of information of the study was shown in supplementary The CONSORT checklist.

**Table 1:** Baseline characteristics of the participants.

	**Total (*n* = 106)**	**Standard training group (*n* = 58)**	**Enhanced training group (*n* = 48)**	***P***
Number of clusters (community healthcare centers)	36	20	16	-
Average cluster size, persons, median (25th, 75th percentile)	3 (2, 4)	3 (1, 4)	3 (2, 4)	0.845
Location^a^				0.143
Fangshan District	58 (54.7)	28 (48.3)	30 (62.5)	
Fengtai District	48 (45.3)	30 (51.7)	18 (37.5)	
Age^b^, years, mean (standard deviation)	37.5 (7.6)	35.5 (6.9)	39.8 (7.8)	0.004
Sex^a^				0.372
Male	35 (33.0)	17 (29.3)	18 (37.5)	
Female	71 (67.0)	41 (70.7)	30 (62.5)	
Occupation^a^				0.764
Physician	78 (73.6)	42 (72.4)	36 (75.0)	
Nurse	28 (26.4)	16 (27.6)	12 (25.0)	
Education^a^, ^c^				0.473
Junior college or lower	33 (33.3)	17 (30.4)	16 (37.2)	
Bachelor or above	66 (66.6)	39 (69.6)	27 (62.8)	
Years of work^d^, median (25th, 75th percentile)	10 (6, 16)	8.5 (6, 15)	10 (5, 18)	0.519

a *Data are expressed as counts (percentages)*.

b *Six participants (4 from the standard and 2 from the enhanced training group) did not report*.

c *Seven participants (2 from the standard and 5 from the enhanced training group) did not report*.

d *Ten participants (4 from the standard and 6 from the enhanced training group) did not report*.

### Comparison of Changes in Dementia-Related Knowledge and Attitudes

The knowledge score at the end of the intervention was significantly higher than that at baseline in each group (*P* both < 0.001). However, the change in the knowledge score between baseline and the end of the intervention in the enhanced training group was similar to that in the standard training group (4.0 vs. 3.5, *P* = 0.218) (Table 2).

**Table 2:** Changes in knowledge, attitudes, and provided service related to dementia detection from baseline to the end of the intervention.

	**Baseline**		**End of the intervention**		**Difference between baseline and the end of the intervention**		**Comparison of the change between two groups**	
	**Standard training group (*n* = 58)**	**Enhanced training group (*n* = 48)**	**Standard training group**	**Enhanced training group**	**Standard training group**	**Enhanced training group**	***P^c^***	***P^d^***
Knowledge score, points^a^	32 (29, 35)	31 (28, 34)	37 (32, 38)	36 (33, 38)	3.5 (−0.3, 7.3)	4.0 (2.0, 7.0)	0.293	0.218
Theoretical knowledge	21 (18, 23)	21 (18, 23)	24 (23, 25)	25 (23, 27)	3.0 (1.0, 5.0)	4.0 (2.3, 5.0)	0.085	0.098
Practical knowledge	11 (10, 12)	11 (10, 12)	13 (9, 14)	11 (10, 12)	1.0 (−2.3, 3.0)	0.0 (−1.0, 1.0)	0.845	0.847
Attitude score, points^a^	8 (8, 9)	8 (7, 9)	9 (7, 9)	8 (8, 9)	0.0 (−1.0, 1.0)	0.0 (−1.0, 1.0)	0.872	0.675
Self-reported dementia screening or referral service in the past month^b^							0.028	0.045
No	44 (75.9)	41 (85.4)	40 (69.0)	26 (54.2)	-	-		
Yes	14 (24.1)	7 (14.6)	18 (31.0)	22 (45.8)	-	-		
Proportion of participants submitting dementia screening records at the end of the study^b^							0.129	0.028
No	-	-	34 (58.6)	21 (43.7)	-	-		
Yes	-	-	24 (41.4)	27 (56.3)	-	-		
Number of submitted records^a^	-	-	15 (6, 46)	27 (11, 55)	-	-	0.138	0.088
Period using records^b^	-	-						
1st−3rd month	-	-	22 (91.7)	27 (100.0)	-	-	0.216	0.196
4th−6th month	-	-	3 (12.5)	14 (51.9)	-	-	0.003	0.002

a *Data are expressed as median (25th−75th percentile)*.

b *Data are expressed as counts (percentages)*.

c *Unadjusted*.

d *Adjusted for age*.

The attitude score of participants in both groups remained positive and stable after the intervention, and no significant difference in the change in attitude score between the two groups was found (*P* = 0.675) (Table 2).

### Comparison of the Change in Provided Service and Submitted Records

Compared with baseline, both groups reported an increase in dementia screening and referral services after the intervention, especially the enhanced training group [24.1% to 31.0% in the standard training group (*P* = 0.302) and 14.6% to 45.8% in the enhanced training group (*P* = 0.003)]. The increase in the enhanced training group was higher than that in the standard training group (*P* = 0.045), although the difference was close to non significant (Table 2).

At the end of the trial, a total of 51 (48.1%) participants submitted 1,845 copies of dementia screening and referral records. The participants in the enhanced training group were more likely to use records than those in the standard training group (*P* = 0.028). Additionally, the median number of submitted records in the enhanced training group was greater than that in the standard training group, although the difference was non significant (Table 2).

Among those participants who submitted records, the participants in each group had a high tendency to use the records (91.7% in the standard training group and 100% in the enhanced training group) during the first 3 months of the study period, and no difference was found between the two groups. In contrast, the proportion of participants who used records during the later 3 months in the enhanced training group was significantly higher than that in the standard training group (*P* = 0.002), although both proportions significantly decreased compared with those during the first 3 months (both *P* < 0.001) (Table 2). Additionally, during the online support period, the questions asked by both the training groups were mainly focused on referring the suspected cases for further assessment and diagnosis.

### Comparison of Dementia Screening and Referral Skills

Except for the CDT, the completion rates of the AD8, referral suggestions and whole record in the enhanced training group were significantly higher than those in the standard training group (Table 3).

**Table 3:** Comparison of the dementia detection skills between the standard and the enhanced training groups according to submitted records^a^.

	**Standard training group (*n* = 661)**	**Enhanced training group (*n* = 1184)**	***P***
AD8^b^			
Not complete	272 (41.1)	216 (18.2)	<0.001
Complete	389 (58.9)	968 (81.8)	
Correct	383 (98.5)	958 (99.0)	0.432
Wrong	6 (1.5)	10 (1.0)	
Clock drawing test			
Not complete	144 (21.8)	287 (24.2)	0.232
Complete	517 (78.2)	897 (75.8)	
Correct	391 (75.6)	760 (84.7)	<0.001
Wrong	126 (24.4)	137 (15.3)	
Referral suggestions			
Not complete	318 (48.1)	396 (33.4)	<0.001
Complete	343 (51.9)	788 (66.6)	
Correct	160 (63.7)	586 (82.8)	<0.001
Wrong	91 (36.3)	122 (17.2)	
Total			
Not complete	440 (66.6)	583 (49.2)	<0.001
Complete	221 (33.4)	601 (50.8)	
Correct	113 (51.1)	452 (75.2)	<0.001
Wrong	108 (48.9)	149 (24.8)	

a *Data are expressed as counts (percentages)*.

b *AD8: Eight-item Interview to Differentiate Aging and Dementia*.

According to those completed records, except for the AD8, the accuracies of CDT, referral suggestions and the whole record in the enhanced training group were significantly higher than those in the standard training group (Table 3).

### Satisfaction and Reaction at the End of the Intervention

A total of 23 (39.7%) participants in the standard training group and 17 (35.4%) participants in the enhanced training group completed reaction and satisfaction surveys at the end of the study. Most participants considered the program efficient and useful for their practice (82.4% in the enhanced training group and 95.7% in the standard training group). Most of the participants said the lectures were easy-to-understand (94.1% in the enhanced training group and 91.3% in the standard training group). The most common suggestion for improving the training content was to introduce more dementia cases in detail in an easy-to-understand way. The participants in both groups indicated the need to add interaction and communication with the trainers during practice and mentioned video-based or online training as a supplement to face-to-face supervision. The participants in the standard training group, rather than the enhanced training group, emphasized the need to learn more about dementia diagnosis, treatment and care skills. No harm or unintended effect was reported during the intervention.

The familiarity between the residents and PCPs and the large aging population in communities were considered advantages for dementia detection, whereas the lower awareness of residents of dementia and the participants' busy clinical practice were the main barriers. Related policy support or incentives from the administration were considered necessary for the implementation of dementia detection.

## Discussion

In this cluster randomized trial, we found that the 3-month standard training and the 6-month enhanced training both had significant effects on improving PCPs' dementia detection-related knowledge and screening as well as referral services. Additionally, the improvement of the service in the enhanced training group was greater than that in the standard training group. Our results suggested that the participants' attitudes toward dementia detection were quite positive before training and remained stable after training. Enhanced training tended to improve the use of dementia screening and referral records. Compared with the standard training group, the enhanced training group had a significantly higher completion rate of the AD8 and referral suggestions and higher accuracy of CDT and referral suggestions. Most participants in both groups considered the training easy-to-understand and useful for their practice. Interaction during practice and online training may be an effective supplement to face-to-face supervision.

In this study, we found that PCPs' knowledge regarding dementia detection at the end of the intervention in each group improved significantly compared with that before training. Likewise, in France, a 2 h group educational meeting supporting the use of brief neuropsychological tools was associated with greater confidence in dementia diagnosis among clinicians (18). Shaji et al. (22) and Ramos-Cerqueira et al. (23) reported that community health workers in India and Brazil could identify dementia patients with reasonable accuracy after a few hours of training. These results clearly indicated that delivering appropriate training for PCPs may serve as an effective modality to enhance their competency regarding dementia detection. Moreover, our findings suggested that PCPs in each group provided more dementia detection services after training, especially the enhanced training group. Similarly, evidence from a cluster randomized trial in the UK showed that decision support systems and workshop formats are effective in improving dementia detection in primary care but not a CD-ROM tutorial compared with the standard training group that received no training intervention (17). However, the study from France showed that a 2 h group educational meeting was not associated with an overall increase in newly identified dementia cases (18). These conflicting results of service change among different training programs may be attributed to the differences in training content, duration, mode of delivery and support. The variety of the participants' professional backgrounds and their clinical settings may also be important reasons for these inconsistent results. Additionally, our results indicated that the enhanced training group provided more services and had better skills related to dementia detection than the standard training group, which suggests that sustainable supervision and support may be required to ensure continuous service and to master skills related to dementia detection.

Although we found that most participants in both groups knew more about and had a positive attitude toward dementia detection at the end of the intervention, our results also showed that nearly 60% of the participants did not provide dementia screening or referral services in the month before the end of the study. Given that the use of a self-administered questionnaire survey may result in inaccurate reports on changes in practice (24), the present study additionally used dementia screening records to evaluate the effect of training on practice. Similarly, we found that approximately half of the participants probably did not use the dementia screening and referral record during the entire study period. Accordingly, Wang et al. (16) reported that basic knowledge and positive attitudes may not ensure health professionals' demonstration of a person-centered approach in dementia care. This inconsistency among knowledge, attitude and related service may imply that the implementation of dementia detection in primary care probably requires more support or conditions, in addition to the improvement of PCPs' related competency. Surr et al. (14) argued that health and social care workforce education should be conceptualized as a complex system, and the individual, meso-, and macrolevels must be considered in understanding learning processes. The feedback of the participants also suggested that enhancing the public's awareness of dementia, modifying PCPs' clinical practice, and providing related support from the administration were important for dementia detection. Of note, the scale and number of older adults served by each included center may not influence the improvement of the participants' skills and dementia detection service, due to CHCs' responsibility for primary health care and the lack of dementia detection service in CHCs in China.

Regarding the training method adopted, previous studies (14–18) used a variety of delivery methods, although predominantly face-to-face learning was often employed alongside other methods. Learning through a written resource (either hard copy or online) or purely classroom-based training consistently yielded no or weak effects on knowledge gains, whereas a combination of theory (through classroom-based learning) and practice (in-service learning) was more likely to produce positive results in improving staff confidence, competency or self-efficacy (14). The adoption of a specific tool or structured method may strongly support participants in adapting their behavior and changing their practice (14). The present program not only provided didactic lectures and exercises but also involved monthly face-to-face supervision, online support, and screening toolkits, which supported the participants in translating what was learned in the classroom to dementia detection practice. The results of our study verified that multiple training methods may be required with respect to dementia detection training. Smith (25) argued that learners had a reduced requirement for a proximal guide/facilitator and a greater need for interaction and construction as their expertise grew. The participants in our study similarly highlighted the need for interaction and communication with trainers during practice.

### Strengths and Limitations

Rather than comparing training with no training, we compared two different training groups against each other. Our results provide evidence for understanding which program is effective and optimal for delivering dementia detection training programs in primary care. Moreover, we used parallel groups, and cluster randomized design in each research district, which minimized contamination and the potential impact of different districts and maximized the comparability of the two groups. Additionally, this study combined didactic lectures, exercises, monthly face-to-face supervision, online support with social media and screening toolkits targeted at improving PCPs' competency and service in dementia detection. The multiple facets of training methods adopted in this study may provide a meaningful reference for other similar education programs in this informational era.

This study has several limitations. First, the participants were not randomly selected from those who provided services to older adults in enrolled CHCs, and they may not be representative of those who chose not to participate in the study. The present study was conducted in Beijing, a first-tier city in China. The participants' professional background and the supporting conditions in their clinical practice may differ from those of PCPs in less developed regions. The sample included in the analysis was smaller than expected. In brief, multicenter trials, especially those in rural areas, may be needed before generalizing the training program to diverse areas. Second, this study explored the possible advantages and disadvantages of implementing dementia detection in primary care according to the participants' feedback, and the questions asked by the participants during the online support period were not quantitatively recorded. The development of an effective way to motivate the participants to translate their knowledge of and attitudes toward dementia detection into dementia detection practice warrants further study. Third, due to limited resources, the perspective of the elderly individuals who received dementia detection services and the potential impact of the improvement in the participants' dementia detection competency on older adults' health outcomes were not evaluated in the present study. Forth, the drop-out rate in this trial was relatively high, especially the enhanced training group, due to the schedule conflict between their routine work and the training program. Fifth, regarding the relatively small sample size and high drop-out rate, those, who completed the assessments both at baseline and at the end of the intervention, were included in the final analysis, rather than all of those who were randomly assigned to the two groups.

## Conclusion

This study indicated that most PCPs recognized the importance of dementia detection, and that 3 months of standard training may be sufficient to improve their knowledge, whereas 6 months of enhanced training was better on the continuous practice and mastery of skills related to dementia detection. The implementation of dementia detection during primary care practice may also require support from the awareness of residents, in addition to adequate competency of practitioners. This trial may serve as a reference for researchers, clinical educators, and policy specialists to inform the development of PCPs' education in the dementia detection sector, with the aim of improving dementia service.

## Data Availability Statement

The raw data supporting the conclusions of this article will be made available by the authors, without undue reservation.

## Ethics Statement

The studies involving human participants were reviewed and approved by the Ethics Committee of Peking University Sixth Hospital. The patients/participants provided their written informed consent to participate in this study.

## Author Contributions

XL, XY, and HW designed the study. XL, MZ, TL, HZ, CP, ZL, NZ, and HW designed and implemented the intervention and collected and managed the data. XL and MZ scored the clock drawing test. XL and CY performed the statistical analyses. XL drafted the manuscript. All the authors had full access to all the data, contributed to the interpretation of the results, provided intellectual input to the manuscript and approved the final version of the manuscript.

## Conflict of Interest

The authors declare that the research was conducted in the absence of any commercial or financial relationships that could be construed as a potential conflict of interest.

## Publisher's Note

All claims expressed in this article are solely those of the authors and do not necessarily represent those of their affiliated organizations, or those of the publisher, the editors and the reviewers. Any product that may be evaluated in this article, or claim that may be made by its manufacturer, is not guaranteed or endorsed by the publisher.

## Abbreviations

PCPs: primary care providers
CHCs: community healthcare centers
AD8: Eight-item Interview to Differentiate Aging and Dementia
CDT: Clock Drawing Test.
