# Supplementary material for: Effects of an Enhanced Training on Primary Care Providers Knowledge, Attitudes, Service and Skills of Dementia Detection: A Cluster Randomized Trial
Source: Front Neurol. 2021 Jul 23;12:651826. doi: 10.3389/fneur.2021.651826 (PMC8342805; doi:10.3389/fneur.2021.651826)
Supplement: Supplementary file 1 [file Table_1.DOCX]

According to the checklist of CONSORT statement for the reporting of cluster randomized trial (Campbell MK, Piaggio G, Elbourne DR, Altman DG; CONSORT Group. Consort 2010 statement: extension to cluster randomised trials. BMJ. 2012 Sep 4;345:e5661. doi: 10.1136/bmj.e5661. PMID: 22951546.), the CONSORT 2010 checklist of the study “Effects of an enhanced training on primary care provider knowledge, attitudes, service and skills of dementia detection: A cluster randomised trial” was shown as the below.

**Table s1. CONSORT 2010 checklist of information of the study**

| **Section/topic and item No** | **Standard checklist item** | **Extension for cluster designs** | **Page Number** |
| --- | --- | --- | --- |
| **Title and abstract** |  |  |  |
| 1a | Identification as a randomised trial in the title | Identification as a cluster randomised trial in the title | Page 1, line 2 |
| 1b | Structured summary of trial design,methods, results, and conclusions See table 2  (for specific guidance see CONSORT for abstracts) |  | Page 2, details were shown in table s2 |
| **Introduction** |  |  |  |
| Background and objectives: |  |  |  |
| 2a | Scientific background and explanation of rationale | Rationale for using a cluster design | Page 4, line 86-88. |
| 2b | Specific objectives or hypotheses | Whether objectives pertain to the cluster level, the  individual participant level, or both | Page 4-5, line 88-90 |
| **Methods** |  |  |  |
| Trial design: |  |  |  |
| 3a | Description of trial design (such as parallel, factorial) including  allocation ratio | Definition of cluster and description of how the design  features apply to the clusters | Page 5, line 94, line 102 |
| 3b | Important changes to methods after trial commencement (such as  eligibility criteria), with reasons |  | Not applicable |
| Participants: |  |  |  |
| 4a | Eligibility criteria for participants | Eligibility criteria for clusters | Page 5-6, line 100-101, line 118-125 |
| 4b | Settings and locations where the data were collected |  | Page 5, line 94-95 |
| Interventions: |  |  |  |
| 5 | The interventions for each group with sufficient details to allow  replication, including how and when they were actually administered | Whether interventions pertain to the cluster level, the  individual participant level, or both | Page 6-7, line 136-170 |
| Outcomes: |  |  |  |
| 6a | Completely defined prespecified primary and secondary outcome  measures, including how and when they were assessed | Whether outcome measures pertain to the cluster  level, the individual participant level, or both | Page 7-8, line 172-212 |
| 6b | Any changes to trial outcomes after the trial commenced, with  reasons |  | Not applicable |
| Sample size: |  |  |  |
| 7a | How sample size was determined | Method of calculation, number of clusters(s) (and  whether equal or unequal cluster sizes are assumed),  cluster size, a coefficient of intracluster correlation  (ICC or *k*), and an indication of its uncertainty | Page 8-9, line 214-220 |
| 7b | When applicable, explanation of any interim analyses and stopping  guidelines |  | Not applicable |
| **Randomisation** |  |  |  |
| Sequence generation: |  |  |  |
| 8a | Method used to generate the random allocation sequence |  | Page 5, line 103 |
| 8b | Type of randomisation; details of any restriction (such as blocking | Details of stratification or matching if used  and block size) | Page 5, line 94, line 102-103  No stratification was used. |
| Allocation concealment  mechanism: |  |  |  |
| 9 | Mechanism used to implement the random allocation sequence  (such as sequentially numbered containers), describing any steps  taken to conceal the sequence until interventions were assigned | Specification that allocation was based on clusters  rather than individuals and whether allocation  concealment (if any) was at the cluster level, the  individual participant level, or both | Page 5, line 110-111 |
| Implementation: |  |  |  |
| 10 | Who generated the random allocation sequence, who enrolled | Replaced by 10a, 10b, and 10c  participants, and who assigned participants to interventions |  |
| 10a |  | Who generated the random allocation sequence, who  enrolled clusters, and who assigned clusters to  interventions | Page 5-7, line 98-100, line 102-104 |
| 10b |  | Mechanism by which individual participants were  included in clusters for the purposes of the trial (such  as complete enumeration, random sampling) | Page 5, line 116-118 |
| 10c |  | From whom consent was sought (representatives of  the cluster, or individual cluster members, or both)  and whether consent was sought before or after  randomisation | Page 5, line 113-114 |
| Blinding: |  |  |  |
| 11a | If done, who was blinded after assignment to interventions (for  example, participants, care providers, those assessing outcomes)  and how |  | Page 5, line 110-111 |
| 11b | If relevant, description of the similarity of interventions |  | Not applicable |
| Statistical methods: |  |  |  |
| 12a | Statistical methods used to compare groups for primary and secondary outcomes | How clustering was taken into account | Page 9-10, line 240-251 |
| 12b | Methods for additional analyses, such as subgroup analyses and  adjusted analyses |  | Page 9, line 251-252 |
| **Results** |  |  |  |
| Participant flow (a diagram is  strongly recommended): |  |  |  |
| 13a | For each group, the numbers of participants who were randomly  assigned, received intended treatment, and were analysed for the  primary outcome | For each group, the numbers of clusters that were  randomly assigned, received intended treatment, and  were analysed for the primary outcome | Page 10, line 259-269, Figure 1 |
| 13b | For each group, losses and exclusions after randomisation, together  with reasons | For each group, losses and exclusions for both  clusters and individual cluster members | Page 10, line 263-269, Figure 1 |
| Recruitment: |  |  |  |
| 14a | Dates defining the periods of recruitment and follow-up |  | Page 10, line 258 |
| 14b | Why the trial ended or was stopped |  | Page 10, line 258-259 |
| Baseline data: |  |  |  |
| 15 | A table showing baseline demographic and clinical characteristics  for each group | Baseline characteristics for the individual and cluster  levels as applicable for each group | Page 10, line 270-277, Table 1 |
| Numbers analysed: |  |  |  |
| 16 | For each group, number of participants (denominator) included in  each analysis and whether the analysis was by original assigned  groups | For each group, number of clusters included in each  analysis | Page 10, line 267-269 |
| Outcomes and estimation: |  |  |  |
| 17a | For each primary and secondary outcome, results for each group,  and the estimated effect size and its precision (such as 95%  confidence interval) | Results at the individual or cluster level as applicable  and a coefficient of intracluster correlation (ICC or *k*)  for each primary outcome | Page 11-12, line 279-315, Table 2 and 3  ICC was not calculated for the relatively small size of each cluster |
| 17b | For binary outcomes, presentation of both absolute and relative  effect sizes is recommended |  | Table 2 |
| Ancillary analyses: |  |  |  |
| 18 | Results of any other analyses performed, including subgroup  analyses and adjusted analyses, distinguishing prespecified from  exploratory |  | Page 12, line 317-334 |
| Harms: |  |  |  |
| 19 | All important harms or unintended effects in each group (for specific  guidance see CONSORT for harms106) |  | Page 12, line 329 |
| **Discussion** |  |  |  |
| Limitations: |  |  |  |
| 20 | Trial limitations, addressing sources of potential bias, imprecision,  and, if relevant, multiplicity of analyses |  | Page 15, line 425-432, line 441-446 |
| Generalisability: |  |  |  |
| 21 | Generalisability (external validity, applicability) of the trial findings | Generalisability to clusters and/or individual  participants (as relevant) | Page 15, line 415-417, line 422-424, line 425-432 |
| Interpretation: |  |  |  |
| 22 | Interpretation consistent with results, balancing benefits and harms,  and considering other relevant evidence |  | Page 16, 449-457 |
| **Other information** |  |  |  |
| Registration: |  |  |  |
| 23 | Registration number and name of trial registry |  | Page 2, line 54 |
| Protocol: |  |  |  |
| 24 | Where the full trial protocol can be accessed, if available |  | Not applicable |
| Funding: |  |  |  |
| 25 | Sources of funding and other support (such as supply of drugs), role  of funders |  | Page 17, line 473-478 |

**Table s2. Extension of CONSORT for abstracts to reports of cluster randomised trials**

| **Item** | **Standard checklist item** | **Extension for cluster designs** | **Page Number** |
| --- | --- | --- | --- |
| Title | Identification of study as randomised | Identification of study as cluster randomised | Page 1, line 2 |
| Trial design | Description of the trial design (for example, parallel, cluster, non-inferiority) |  | Page 2, line 28-31 |
| **Methods:** |  |  |  |
| Participants | Eligibility criteria for participants and the settings where the data were | Eligibility criteria for clusters  collected | Page 2, line 28-32 |
| Interventions | Interventions intended for each group |  | Page 2, line 32-35 |
| Objective | Specific objective or hypothesis | Whether objective or hypothesis pertains to the cluster level,  the individual participant level, or both | Page 2, line 25-27 |
| Outcome | Clearly defined primary outcome for this report | Whether the primary outcome pertains to the cluster level,  the individual participant level or both | Page 2, line 36-38 |
| Randomisation | How participants were allocated to interventions | How clusters were allocated to interventions | Page 2, line 29-31 |
| Blinding (masking) | Whether or not participants, care givers, and those assessing the outcomes  were blinded to group assignment |  | Page 2, line 35-36 |
| **Results:** |  |  |  |
| Numbers randomised | Number of participants randomised to each group | Number of clusters randomised to each group | Page 2, line 39-40 |
| Recruitment | Trial status |  | Page 3, line 55 |
| Numbers analysed | Number of participants analysed in each group | Number of clusters analysed in each group | Page 2, line 40-42 |
| Outcome | For the primary outcome, a result for each group and the estimated effect  size and its precision | Results at the cluster or individual level as applicable for each  primary outcome | Page 2, line 42-50 |
| Harms | Important adverse events or side effects |  | Due to little harm reported and word limit of the abstract, this item was not reported in abstract |
| **Conclusions** | General interpretation of the results |  | Page 2, line 51-53 |
| **Trial registration Registration** | number and name of trial register |  | Page 2, line 54 |
| **Funding** | Source of funding |  | Due to word limit of the abstract, this item was not reported in abstract |
