# Supplementary material for: Effects of an Enhanced Training on Primary Care Providers Knowledge, Attitudes, Service and Skills of Dementia Detection: A Cluster Randomized Trial
Source: Front Neurol. 2021 Jul 23;12:651826. doi: 10.3389/fneur.2021.651826 (PMC8342805; doi:10.3389/fneur.2021.651826)
Supplement: Supplementary file 2 [file Table_2.DOCX]

**What do you know about dementia?**

*(Please answer all the questions one by one. If you have any question, please ask our research staff.)*

**A. Personal Information**

1. Affiliation: District Community Health Center
2. Name:
3. Gender: 1 Female 2 Male
4. Date of birth: / / (dd/mm/yyyy)
5. Working years as a general practitioner:
6. Clinical practice sessions in a week(Multiple choice):

| 1 Monday morning | 2 Monday afternoon | 3 Tuesday morning | 4 Tuesday afternoon |
| --- | --- | --- | --- |
| 5 Wednesday morning | 6 Wednesday afternoon | 7 Thursday morning | 8 Thursday afternoon |
| 9 Friday morning | 10 Friday afternoon | 11 Saturday morning | 12 Saturday afternoon |
| 13 Sunday morning | 14. Sunday afternoon |  |  |

1. Professional title:

| 1 House physician | 2 Doctor in charge | 3 Assistant director physician |
| --- | --- | --- |
| 4 Director physician | 5 Other, please specify: | |

1. Post in the health center:
2. Education:

| 1 Junior high school | 2 High school | 3 Junior college |
| --- | --- | --- |
| 4 Bachelor degree | 5 Masters degree | 6 Doctorate |

1. Contacts:

1 Office phone:

2 Mobile phone:

3 WeChat ID:

**B. Please read each statement carefully and tick the box if you think the statement is "True" or "False".**

| **No.** | **Statement** | **True** | **False** |
| --- | --- | --- | --- |
| **1** | Dementia is one of the main diseases that cause disability and loss of independence in daily life for older adults. |  |  |
| **2** | All patients with mild cognitive impairment will develop dementia finally. |  |  |
| **3** | Vascular dementia is the leading cause of dementia. |  |  |
| **4** | Alzheimer's disease is a fatal and cerebral-neuro degenerative disease. |  |  |
| **5** | Ageing is the most important risk factor for Alzheimer's disease. The risk of developing Alzheimer's disease increases with ageing. |  |  |
| **6** | The declined ability of activities of daily life is one of the early symptoms of dementia. |  |  |
| **7** | The course of dementia includes the mild, moderate and severe stage. |  |  |
| **8** | Dementia only affects patients' memory, thinking, and other cognitive fuction, and does not affect physical function. |  |  |
| **9** | Diagnosis and treatment of dementia should be carried out in the memory clinic in a higher level hospital. |  |  |
| **10** | Dementia patients at an early stage can take the medicine by themselves, and there is no need to seek professional help. |  |  |
| **11** | Alzheimer's disease is curable by medicines. |  |  |
| **12** | Currently, the medication approved by the State Food and Drug Administration for the treatment of Alzheimer's disease is only the cholinesterase inhibitor. |  |  |
| **13** | People with dementia need to be referred to a specific institute only when they appear severe cognitive impairment. |  |  |

**C. Please choose the correct answer for each question. Please notice that all questions are single choice, except for additional instructions.**

1. Dementia is caused by which leads to cognitive decline.

| 1. Brain degeneration | 2. Physical function degeneration |
| --- | --- |
| 3. Memory loss | 4. Intelligent degeneration |

1. In the following options, which is a common symptom of dementia?

| 1. Difficulties in learning | 2. Difficulties at work |
| --- | --- |
| 3. Difficulty in activities of daily living | 4. All above |

1. In the following options, which is not a symptom of dementia?

| 1 Loss of the recent memory, affecting the working ability |
| --- |
| 2. Time and place disorientation |
| 3. Changes in mood or behavior |
| 4. Talking to oneself |

1. What is the difference between mild cognitive impairment and dementia?

| 1. Comparable cognitive decline |
| --- |
| 2. Both have severe behavioral problems |
| 3. Only patients with dementia have emotional problems |
| 4. Patients with mild cognitive impairment do not have function impairment |

1. In the following options, which is caused by dementia?

| 1. Cognitive function decline | 2. Insanity | 3. Sudden decline in mobility |
| --- | --- | --- |

1. The recent memory is declined in the early-stage patients with dementia, but their could remain relatively good.

| 1. Judgment | 2. Long-term memory |
| --- | --- |
| 3. Thinking | 4. Reasoning |

1. The cutoff score of the AD-8 is ___________.

| 1. 2 points | 2. 3 points | 3. 4 points | 4. 5 points |
| --- | --- | --- | --- |

1. In the following statement about the scoring criteria of the Clock Drawing Test, which is correct? (Multiple choice).

| 1. If drawing a closed circle, then get 1 point. |
| --- |
| 2. If the position of the figure is accurate, then get 1 point. |
| 3. If none of the 12 numbers missed, then get 1 point. |
| 4. If the position of the pointer is accurate, then get 1 point. |

1. A score of _______ of the Clock Drawing Test indicates cognitive decline.

| 1. 3-4 points | 2. 1-2 points |
| --- | --- |

1. People who would benefit from the early diagnosis of dementia are (Multiple choice).

| 1. Patients | 2. Family members/Caregivers | 3. Health policymakers |
| --- | --- | --- |

1. The ___________ should be considered first when treating the behavioral problems in patients with dementia.

| 1. Pharmacotherapy | 2. To relieve stress | 3. Non-pharmacotherapy |
| --- | --- | --- |

1. In the following statements, which is not highlighted in the standardized treatment procedure for dementia?

| 1. Scientific medicine treatment |
| --- |
| 2. Systematic social-psychosocial therapy |
| 3. Standardized caregiver support and counselling |
| 4. Regular cognitive training |

1. In the past month, Liang Zhang could concentrate in watching TV, reading magazines, playing cards or chess. But it happened once a week that he forgot where he put his keys or glasses, but could find them within five minutes.
2. Do you think Liang Zhang has any difficulty in concentration or memory? If yes, what severity do you think he is?

| 1. No problem | 2. Mild | 3. Moderate | 4. Severe | 5. Very severe |
| --- | --- | --- | --- | --- |

1. Your advice for him is:

| 1. It is normal with ageing. |
| --- |
| 2. To take the memory screening and being alert to the possibility of the early-stage dementia |
| 3. This may be dementia, and there is no medicine to treat it. |
| 4. This may be dementia, and you should seek help timely for professional institutions |

1. Li Song likes cooking new dishes from the recipe, but she often makes mistakes. Usually, she has to re-read the recipe several times until she finally learns to cook the new dish.

**a**. Do you think Song Li has any difficulty in concentration or memory in the past month? If yes, what severity do you think she is?

| 1. No problem | 2. Mild | 3. Moderate | 4. Severe | 5. Very severe |
| --- | --- | --- | --- | --- |

**b.** Your advice for her is:

| 1. It is normal with ageing. |
| --- |
| 2. To take the memory screening and being alert to the possibility of the early-stage dementia |
| 3. This may be dementia, and there is no medicine to treat it. |
| 4. This may be dementia, and you should seek help timely for professional institutions |

1. In the past month, Wei Li could not concentrate for more than 15 minutes every time. When others speak with him, it was hard for him to pay attention to listen to their words. When he did something, he could not complete it and often forgot what he wanted to do. But when he knew new friends, he could remember their names.

**a**. Do you think Wei Li has any difficulty in concentration or memory? If yes, what severity do you think he is?

| 1. No problem | 2. Mild | 3. Moderate | 4. Severe | 5. Very severe |
| --- | --- | --- | --- | --- |

**b**. Your advice for him is:

| 1. It is normal with ageing. |
| --- |
| 2. To take the memory screening and being alert to the possibility of the early-stage dementia |
| 3. This may be dementia, and there is no medicine to treat it. |
| 4. This may be dementia, and you should seek help timely for professional institutions |

1. For the older patients who have memory problems (e.g. repeatedly asking the same questions, often forgetting things), emotional problems (e.g. emotions become childlike and intense, and change quickly), behavioral problems (e.g. being suspicious, impulsive, irritable, sleep-reversed, wandering at night) or social function problems (e.g. dislike going out, reluctance to contact with others, declined abilities for working or housework), how will you deal with these cases? (Multiple choice)

| 1. Tell them that it is common problems among older adults, and there is no need to worry about them. |
| --- |
| 2. Suggest take the clinical assessment to determine whether they have dementia. |
| 3. Suggest go to the memory clinic in higher level hospital for further diagnosis and treatment. |
| 4. Suggest family members and the live-in care worker take care of the patients, and there is no need to seek professional help. |
| 5. Suggest limit the older adult' outdoor activities to avoid accidents |
| 6. Suggest take memory training and daily life function training and encourage the older adults to take part in social activities |
| 7. Suggest take the pharmacotherapy |

1. What do you think about older patients with dementia who have memory problems, emotional problems, behavioral problems, or social function problems? (Multiple choice)

| 1. They are lengthy, childish, sometimes unable to understand normal rules, and often make people feel irritable and embarrassed. |
| --- |
| 2. It is normal for patients with dementia to have such problems, and others should be tolerant |
| 3. Being patient in explaining these problems and persuading others, and trying to understand that these problems may be an expression of unmet needs, and trying to meet their reasonable needs. |

1. How many older adults with memory problems do you see at the clinic in the last month?

| 1. More than 10 persons (The specific number of people is ) |
| --- |
| 2. 5-10 persons (The specific number is ) |
| 3. 1-4 persons (The specific number is ) |
| 4. None |

1. How many percentages of older adult with memory problems taking the screening in your clinical practice in the last month?

| 1. More than 80% | 2. 50%-80% |
| --- | --- |
| 3. 30%-50% | 4. Less than 30% |

1. How many older adults with memory problems taking the memory screening in your clinical practice in the last month?

| 1. More than 10 persons (The specific number is ) |
| --- |
| 2. 5-10 persons (The specific number is ) |
| 3. 1-4 persons (The specific number is ) |
| 4. None |

1. How many older adults in your clinical practice diagnosed with a high risk of dementia and referred to a higher level hospital in the last month?

| 1. More than 10 persons (The specific number is ) |
| --- |
| 2. 5-10 persons (The specific number is ) |
| 3. 1-4 persons (The specific number is ) |
| 4. None |
